# Tiletamine–Zolazepam Use in Exotic Pets and Wildlife Anesthesia: A Narrative Review Towards Practical Guidelines

**DOI:** 10.3390/ani16091300

**Published:** 2026-04-23

**Authors:** Emmanuel Risi, Romain Potier, Carsten Grøndahl, Laure Poincelot

**Affiliations:** 1 FauneVet, 23 Impasse du pré de l’arche, 44470 Carquefou, France; emmanuel.risi@wanadoo.fr (E.R.); r.potier@faunevet.fr (R.P.); 2 Vet Consult DK, Copenhagen Zoo, Skovvej 58, Charlottenlund, 2920 Copenhagen, Denmark; carsten@vetconsult.dk; 3 Virbac SA, 13ème Rue LID, 06515 Carros, France

**Keywords:** anesthesia, wildlife, exotic, tiletamine, zolazepam, zoletil

## Abstract

This narrative review examines the clinical application of tiletamine–zolazepam (TZ) in exotic pet and wildlife anesthesia, addressing the complexities inherent in managing a broad taxonomic spectrum with diverse physiological profiles and temperaments. As a fixed-dose combination, TZ is a cornerstone of multimodal protocols designed to achieve balanced anesthesia. Its lyophilized formulation permits reconstitution with various sedative solutions, facilitating low-volume administration, a critical requirement for the immobilization of wildlife and small exotic patients. Given the significant variability in species-specific responses and environmental influences, selecting and adapting appropriate TZ-based protocols remain a challenge for practitioners. By synthesizing heterogeneous data into expert-validated guidance, this review provides specialized and general veterinarians with practical considerations for the judicious use of TZ. Emphasis is placed on integrating TZ within multimodal protocols to mitigate arousal risks, ensure consistent immobilization, and facilitate rapid recovery. This approach seeks to optimize anesthetic outcomes and promote animal welfare across these physiologically diverse populations.

## Simple Summary

Anesthetizing exotic pets and wildlife is a significant challenge due to the vast differences in size, biology, and behavior between species, ranging from small rabbits to large animals like bears. This review explores the use of tiletamine–zolazepam, a combination of two drugs working synergistically to maintain animals unconscious and relaxed during medical procedures. The primary goal is to provide veterinarians with a practical guide on using this combination effectively to ensure animal safety and comfort. A major benefit of this medication is its powder form, which can be easily mixed with other drugs to create a balanced approach to anesthesia. When adapted to the specific needs of different animals, the combination of tiletamine and zolazepam provides reliable anesthesia and comfort. These findings are valuable because they help veterinarians provide better medical care for unique species, ultimately improving animal welfare and supporting vital conservation efforts for the world’s wildlife.

## 1. Introduction

Achieving general anesthesia safely remains a significant challenge in veterinary medicine, as no protocol is entirely devoid of risk. Anesthesia typically necessitates a multimodal approach to ensure analgesia, loss of consciousness, muscle relaxation and sedation. These states are ideally achieved through synergistic drug combinations that target various physiological pathways. Regardless of the pharmacological protocol employed, secure airway management via intubation and consistent oxygen supplementation remain the critical pillars of safe anesthetic practice. The optimal balanced anesthetic plan is multifaceted, accounting for species-specific requirements, the veterinarian’s familiarity with the protocol, the pre-anesthetic examination and health status, comprehensive risk assessment, drug availability, cost, and the anticipated duration of the procedure [1]. Despite these established principles, anesthesia in wildlife and exotic species is frequently complicated by a profound lack of standardized pharmacological data. Practitioners often face significant clinical challenges, including extreme taxonomic diversity, varied metabolic rates, and the practical difficulties of monitoring “non-traditional” animals. Among injectable anesthetic agents, the fixed-dose combination of tiletamine–zolazepam (TZ) has become particularly important in exotic and wildlife anesthesia due to its capacity to produce a balanced anesthetic state. The TZ-based combination is commonly utilized alongside other agents to enhance analgesia, loss of consciousness, muscle relaxation and sedation. Moreover, the fixed-dose combination of TZ is a cornerstone of injectable anesthesia thanks to its volume efficiency. However, the effects of TZ-based protocols can be highly unpredictable across different families and genera. This inter-taxa variability often leads to clinical uncertainty for the general practitioner and presents a complex pharmacological puzzle for the specialist. To address this need in the field, this narrative review synthesizes a vast and fragmented body of bibliographic data, critically analyzed through the collective clinical expertise and field experience of the co-authors. By consolidating diverse literature into actionable guidance, this work aims to provide specialized practitioners with refined protocols while offering a practical roadmap for general practitioners faced with uncommon or emergency cases. Ultimately, this review serves as a comprehensive resource for the judicious use of TZ-based protocols across a broad range of species.

## 2. Methods

The methodology for this narrative review was designed to provide a rigorous and clinically relevant synthesis of TZ applications across the diverse spectrum of exotic pet and wildlife anesthesia. Primary data acquisition was performed through a systematic search of the PubMed and Web of Science databases, encompassing literature published between 1980 and 2025. This electronic search was augmented by targeted manual reviews of prominent specialized journals, including the *Journal of Exotic Pet Medicine*, the *Journal of Herpetological Medicine and Surgery*, the *Journal of Avian Medicine and Surgery*, *Veterinary Clinics of North America: Exotic Animal Practice*, the *Journal of Zoo and Wildlife Medicine*, the *Journal of Wildlife Diseases*, *Veterinary Anaesthesia and Analgesia*, and the *Journal of Wildlife Management*. The search strategy utilized Boolean operators to intersect core pharmacological agents, “Zoletil”, “tiletamine” AND “zolazepam”, with various descriptors such as “anaesthesia” to ensure a comprehensive capture of relevant pharmacological and clinical data. To achieve granular coverage across diverse biological classes, secondary targeted searches were executed using specific taxonomic descriptors. These included, but were not restricted to: “rabbit”, “rodent”, “ferret”, “bird”, “reptile”, “snake”, “lizard”, “tortoise”, “turtle”, “exotic pets”, “primate”, “tiger”, *Panthera*, “bear”, “*Ursus*”, “*Sus*”, “bison”, “avian”, and “wildlife”. The synthesized results represent the consolidated output of these multi-database and manual searches. From an initial yield of approximately 750 records, 104 studies were ultimately selected for inclusion based on their clinical significance and methodological quality. Literature selection was governed by the principles of evidence-based veterinary medicine. Priority was accorded to peer-reviewed original research, prospective clinical trials, and retrospective studies that documented objective physiological parameters and standardized induction and recovery intervals. Conversely, anecdotal reports lacking precise dosages or monitored clinical data were excluded, as were studies primarily focused on domestic canids or felids, unless they provided vital comparative pharmacological context. While the narrative format of this review prioritized clinical utility over a formal meta-analysis, the search strategy followed structured principles to ensure transparency and reproducibility. This evidence base was further fortified by consulting supplementary sources, including veterinary textbooks, doctoral theses, and conference proceedings specialized in non-traditional species. Recognizing the inherent limitations of wildlife research, where large-scale randomized controlled trials are often precluded by ethical, logistical, or conservation constraints, the strength of the clinical guidance was carefully calibrated against the quality of available evidence. In instances where the published literature was fragmented, the authors’ collective clinical expertise in zoo, exotic pet, and wildlife anesthesia was integrated to bridge the gap between theoretical data and practical application. To maintain a rigorous distinction between evidence-based synthesis and expert extrapolation, all recommendations derived from clinical experience are systematically signposted throughout the manuscript using qualifying phrases such as “based on the authors’ clinical experience.” This approach ensures a balanced and actionable framework for the use of TZ across a wide array of non-domestic taxa. The methodology and findings of this review should be interpreted in light of several limitations inherent to its narrative design. Although a structured literature search was performed, this study does not adhere to a formal systematic review protocol, and no meta-analysis was conducted. Consequently, the selection of studies may be susceptible to selection bias, and the quality of evidence varies across the included publications. Furthermore, the available literature regarding TZ use in exotic and zoo species is highly heterogeneous, with significant variability in study designs, sample sizes, target species, and reported outcome measures. This diversity precludes direct comparisons between studies and restricts the ability to establish universally standardized protocols. For several taxa, specifically reptiles, birds, and less commonly studied mammals, available data remain sparse or are derived from small case series. In these instances, clinical recommendations rely partially on the authors’ professional experience, which may introduce an element of subjective interpretation. Additionally, extrinsic factors such as environmental conditions, handling techniques, and clinical settings (e.g., field versus hospital environments) may significantly influence anesthetic outcomes, further limiting the generalizability of the findings. This approach ensures a balanced and actionable framework for the use of TZ across a wide array of non-domestic taxa while providing a clinically relevant synthesis to support evidence-informed decision-making.

## 3. Mechanism of Action

The combination of the dissociative anesthetic tiletamine and the benzodiazepine zolazepam creates a balanced anesthetic state due to their synergistic interaction. Tiletamine, a dissociative anesthetic, primarily functions as a non-competitive antagonist of the N-methyl-D-aspartate (NMDA) receptor. By disrupting the binding of the excitatory neurotransmitter glutamate, tiletamine causes a functional dissociation of the cortex from sensory input, simultaneously depressing the limbic, thalamocortical, and reticular activating systems. This action leads to unconsciousness and, consequently, a lack of pain perception [2]. Zolazepam is a benzodiazepine that enhances the effects of the inhibitory neurotransmitter gamma-aminobutyric acid (GABA) by binding to the GABAA receptor [3]. This binding to specific GABAA receptor subtypes allosterically modulates GABAergic neurotransmission. When combined with tiletamine, this action decreases neuronal excitability, resulting in the desired muscle relaxation and sedation [4].

Following either intramuscular (IM) or intravenous (IV) injection, both tiletamine and zolazepam are quickly absorbed into, or distributed within, the systemic circulation. The rate of absorption is influenced by the specific formulation, particularly the presence of solubilizing agents, and the vascularity of the injection site [5,6,7]. Both drugs are highly lipophilic, which enables them to readily cross the blood–brain barrier and enter the central nervous system. Tiletamine exhibits a relatively large volume of distribution, indicating extensive penetration into tissues beyond the bloodstream. Similarly, zolazepam is highly lipophilic and distributes quickly into various tissues, notably adipose tissue, which can act as a reservoir leading to prolonged drug effects [5,7,8]. The pharmacokinetics of the two drugs, specifically their elimination half-life, exhibit considerable variability. This is influenced by factors such as the animal’s species, age, health status, and any concurrent medications, all of which affect the intensity and duration of the resulting anesthesia [5,9]. Both drugs are metabolized primarily in the liver and subsequently excreted mainly by the kidney [8,10]. Consequently, longer duration of anesthesia and recovery times are anticipated in animals suffering from renal disease [5,6,11]. A smaller amount of the administered dose is also eliminated through the biliary system and excreted in the feces.

The TZ combination exerts dose-dependent hemodynamic effects. In dogs and cats, although it does not compromise cardiac output, the administration of 10 mg/kg of TZ intramuscularly may lead to a decrease in cardiac ejection fraction, whereas dosages exceeding 15 mg/kg can induce cardiovascular depression, characterized by reduced myocardial contractility and systemic hypotension [6]. While feline hypertrophic cardiomyopathy is a frequently cited contraindication, there is a notable paucity of data regarding TZ safety across the broad spectrum of heart diseases, particularly in non-domestic species [5,6]. Consequently, the use and dosage of TZ must be evaluated and adapted with caution in any patient where tachycardia or increased sympathetic tone (e.g., dysrhythmias) would be deleterious to prevent an unsustainable increase in myocardial oxygen demand. TZ is also associated with increases in cerebral metabolism, cerebral blood flow and intracranial pressure [6]. The effects of TZ on intraocular pressure remain a subject of debate in canine medicine. Although some studies indicate potential elevations, the clinical significance of such increases has not been definitively established [12,13].

A primary pharmacological characteristic of the TZ combination is its fixed 1:1 ratio. While this formulation is designed to ensure a synergistic balance between dissociative anesthesia and benzodiazepine-mediated muscle relaxation, it presents inherent pharmacokinetic and pharmacodynamic challenges when applied across the diverse metabolic profiles of species. The principal implication of this fixed ratio is the variability in metabolic rates between taxa, which may result in one active ingredient persisting significantly longer than the other. Consequently, the required synergy between the dissociative and sedative components can be compromised, particularly during the recovery phase. However, the current veterinary literature lacks comprehensive, species-specific PK/PD modeling for the vast majority of domestic, exotic and wildlife species, making it unfeasible to evaluate these metabolic divergences for every taxon. In the absence of such data, the clinical use of TZ relies primarily on observed efficacy, the monitoring of anesthetic depth, and the assessment of recovery quality. To mitigate the constraints of the fixed ratio, clinical protocols often leverage the “versatility of reconstitution” by incorporating TZ into multimodal regimens alongside alpha-2 agonists or opioids. This approach facilitates a reduction in the required dosage of each agent while enhancing their synergistic pharmacological effects. Consequently, this strategy narrows the potential window for metabolic divergence and improves the overall quality of the anesthetic event through a balanced anesthesia framework.

## 4. Clinical Application in Exotic Pets

Exotic animals often require sedation or anesthesia for patient examination, diagnostics, and surgery. For relatively smaller species, it is desirable to have a drug that is injectable in small volumes, subcutaneously, intraperitoneally or intramuscularly, due to technical difficulties in performing intravenous catheterization without sedation [14]. Hence, TZ can be useful because its lyophilized formulation allows the solution to be reconstituted at the desired concentration. Some scientific studies on its use in small mammals, birds and reptiles confirm that the levels of immobilization appear to be dependent on the dose, drug combination, and the route of administration.

### 4.1. Dosage and Clinical Application in Rabbits

In rabbits, TZ protocols alone or combined with other drugs have been published (Table 1) [15,16,17]. TZ often provides short action and incomplete anesthesia or analgesia, necessitating the addition of other sedative agents [16,17,18]. Some classical side effects are then observed, including cardiac depression; reduced PaO_2_, pH, and temperature; and increased PaCO_2_ and HCO_3_ [16,19].

For sedation, intranasal (IN) administration of TZ has been successfully tested, alone [20] or in combination with butorphanol [15] (Table 1). Concerns have been raised regarding TZ-associated nephrotoxicity in rabbits, particularly at higher dosages (64 and 32 mg/kg), including increased blood urea nitrogen (BUN), serum creatinine, proteinuria, urinary casts, and histologically confirmed renal tubular necrosis, with evidence suggesting that tiletamine is the primary component responsible for renal toxicity [21,22,23,24]. However, clinical studies provide more nuanced results. Post-anesthesia BUN and urea levels increased significantly with a tiletamine–zolazepam–xylazine (TZX) combination (5 + 15 mg/kg), but not with TZ alone at 15 mg/kg [17]. Other studies have reported no significant changes in renal parameters at similar or lower dosages [16,19]. These discrepancies likely reflect differences in dosage, drug combinations, and experimental conditions, suggesting that nephrotoxicity is dose-dependent and influenced by protocol design. From a clinical perspective, these findings indicate that while TZ-associated nephrotoxicity cannot be disregarded, its occurrence appears limited under standard clinical conditions when appropriate dosages are used. Nevertheless, a cautious and preventive approach is recommended. Preventive fluid therapy should be considered to support renal perfusion, particularly during prolonged procedures or when TZ is combined with other sedative agents such as alpha-2 agonists. In addition, pre- and post-anesthetic evaluation of renal parameters (e.g., BUN and creatinine) is advisable in at-risk patients. Overall, careful dose selection, adequate hydration, and appropriate monitoring are essential to minimize potential renal complications associated with TZ use in rabbits.

### 4.2. Dosage and Clinical Application in Rodents

TZ is commonly used and induces satisfactory anesthesia in laboratory or pet rodents (Table 2). However, experimental studies with TZ alone showed good and effective surgical anesthesia in rats, but not for mice or hamsters at dosages up to 80 mg/kg [25]. Moreover, using TZ alone leaves the ocular, laryngeal, pharyngeal, swallow, and corneal reflexes intact; therefore, an eye lubricant is advised [26].

When used for sedation only, TZ can be administered to mice using voluntary ingestion into cream cheese [27]. A dose of 20 mg/kg led to mild sedation (ataxia, immobility and recumbency) and reduced stress [27].

In the experimental field, some studies have demonstrated that blood parameters in mice and rats exposed to CO_2_ inhalation or a TZX mixture were substantially different [28,29]. The levels of the majority of serum clinical biochemical parameters in rats and mice are overestimated following CO_2_ inhalation [28,29]. Injection of TZX was a more feasible method for terminal blood sampling, which also reduced the suffering and stress of animals (Table 2) [29].

TZX is a suitable anesthetic for blood analysis, showing no interference with glycogen levels or serum markers and no hemolysis [30]. However, side effects like acidosis, hypoxia, and hypercapnia can occur [31]. Unlike ketamine–xylazine or pentobarbital, TZ causes fewer cardiovascular effects in rats, maintaining a higher mean arterial pressure and cardiac index; however, like other anesthetics, it induces dose-dependent hypotension and prolonged recovery [32]. At 80 mg/kg in rats, TZ anesthesia results in higher respiratory rate, blood pressure, and cardiac function indices compared to other protocols [33].

In guinea pigs, short-term chemical restraint without analgesic effects was observed after TZ alone at dosages of 50 or 100 mg/kg [34]. These dosages of TZ did not cause loss of the palpebral reflex, reduction in jaw or muscle tone, or responses to stimuli [14,34].

In gerbils and hamsters, the availability of safe parenteral anesthetics is limited. Some reports give information on the use of TZ, with a preference for the intraperitoneal route versus the IM route (Table 2) [14,35,36,37]. While Forsythe et al. (1992) [36] reported localized muscle lesions in hamsters following IM administration, the clinical significance of these findings remains unclear. Based on the authors’ clinical experience, such injection-site reactions appear to be isolated occurrences and have not been consistently identified as a specific risk associated with any particular species. Numerous studies have reported that TZ alone is not sufficient for efficient analgesia. However, when used as a component of a balanced anesthesia protocol, TZ remains a beneficial combination for inducing safe, rapid, and profound surgical anesthesia (Table 2) [28,29,30,38,39,40,41,42,43,44,45,46,47].

**Table 2:** Use of Tiletamine–Zolazepam in rodents.

Species, Drugs and Dosage	Effects and Comments
Mouse TZ 20–60 mg/kg + D 0.4–0.8 mg/kg IP [39,40]	Variable unreliable anesthesia of 58% of mice Anesthetic duration not altered by increased dosages (ceiling effect reached, no benefit of increasing dosages TZ above 40 mg/kg or D above 0.6 mg/kg) Risk of incidence of dexmedetomidine-induced urethral obstruction (66%)
Mouse TZ 10–40 mg/kg IP + D 0.2–0.6 mg/kg IP + B 3 mg/kg SC [39,40]	Adequate-to-excellent levels of anesthesia of 100%, longer and more reliable duration of anesthesia than D and TZ alone Anesthesia duration of 143 ± 16 min with D 0.2 mg/kg + TZ 40 mg/kg + B 3 mg/kg
Mouse TZ 80 mg/kg + X 20 mg/kg IP [38]	22 ± 2 min mean of induction 30 ± 5 min mean of surgical anesthesia 263 ± 10 min mean of sleeping time 10% of death during surgery (vasectomy), lower than a KX protocol
Rat and Mouse TZ 10–30 mg/kg IP + M 0.03–1 mg/kg SC [14]	
Rat and Mouse TZ 12.5 mg/kg + X 7.5 mg/kg IM [28,29]	Surgical anesthesia after 5–6 min (loss of the righting reflex, pedal reflex, and tail withdrawal after pinching)
Rats TZ 30, 40, 50, 60 mg/kg IP [32]	Dose-dependent increase in duration of anesthesia fewest adverse cardiovascular effects at 40 and 50 mg/kg
Rats Protocol 1 TZ 10 mg/kg + D 0.25 mg/kg) SC Protocol 2 TZ 10 mg/kg + D 0.25 mg/kg + T 12.5 mg/kg SC [46]	Sufficient surgical anesthesia > 45 min Tramadol did not change times to righting or paw withdrawal loss or duration of anesthesia but provided pain control even following atipamezole’s reversal of D
Rats TZ 40 mg/kg IP + M 0.035 mg/kg IM TZ 50 mg/kg IP + M 0.02 mg/kg IM [41]	Deep anesthesia after 5 min No mortality Duration of surgical anesthesia differed statistically (Pedal withdrawal reflex at 1.71 ± 0.07 h and 2.04 ± 0.12 h, respectively) Sleeping time differed statistically (3.35 ± 0.34 h and 2.83 ± 0.31 h, respectively)
Rat TZ 50 mg/kg IM [42,47]	Allows surgical procedure for experimental research
Rat TZ 30 mg/kg IM [31]	Allows blood sampling for experimental research
Rat TZ 50 mg/kg + X 11 mg/kg IM [30]	Quick and deep anesthesia No effect on serum hemolysis or hepatic and muscular glycogenolysis
Rat TZ 15 mg/kg + X 9.3 mg/kg IM, IP [44]	Anesthesia for surgical procedure time to deep anesthesia reduced with IM
Rat TZ 20 or 40 mg/kg IP, IM [25,26,46]	30–60 min of satisfactory surgical anesthesia and analgesia Dose dependent length of anesthesia and somewhat longer in females
Hamster TZ 20 mg/kg + X 10 mg/kg IP [36]	Adequate for restraint purposes No nephrotoxicity
Hamster TZ 30 mg/kg + X 10 mg/kg IP [36]	Safe, reliable level of surgical anesthesia No gross or histopathologic lesions. No nephrotoxicity IM route failed to consistently produce anesthesia and caused gross and histopathologic muscle lesions
Gerbil, Hamster TZ 10–30 mg/kg IP + M 0.1–0.2 mg/kg SC [14]	
Gerbil TZ 60 mg/kg [37]	Safe anesthetic Suitable for major surgical procedures Prolonged recovery time, requiring closely monitoring Lower dosages for less nociceptive and noninvasive manipulations
Gerbil TZ 20 mg/kg + X 10 mg/kg IP [35]	
Chinchilla TZ 11–44 mg/kg IM [9]	115–431 min of surgical anesthesia
Chinchilla TZ 20–40 mg/kg IM [35]	
Guinea pigs TZ 60 mg/kg IP + X 5 mg/kg IP + B 0.1 mg/kg IM [43]	Long-duration deep surgical anesthesia (mean of 66 min) and moderate surgical anesthesia for another 43 min Smooth induction and recovery Decreased respiratory rate and PaO_2_ Increased PaCO_2_ Minor-to-moderate effect on the cardiovascular system
Guinea pigs TZ 10 mg/kg + K 4 mg/kg + X 5 mg/kg + B 0.1 mg/kg IM [45]	Lateral recumbency within 2 min. Cardiorespiratory and blood pressure depression Decreased respiratory rate Longer analgesic duration of 30–40 min suitable for castration and ovariohysterectomy
Guinea pigs TZ 5 mg/kg + K 2 mg/kg + X 2.5 mg/kg + B 0.05 mg/kg IM X reversed with yohimbine 2 mg/kg IM after 100 min [45]	Lateral recumbency within 2 min. Cardiorespiratory and blood pressure depression Decreased respiratory rate

Abbreviations: IM = Intramuscularly, IP = Intraperitoneal, SC = Subcutaneous, TZ = Tiletamine–Zolazepam, D = Dexmedetomidine, B = Butorphanol, X = Xylazine, M = Medetomidine, T = Tramadol, K = Ketamine.

### 4.3. Dosage and Clinical Application in Ferrets

In ferrets, TZ alone provides appropriate depth of anesthesia when compared to that in rodents and rabbits. Both 12 mg/kg and 22 mg/kg doses administered intramuscularly produced dose-dependent immobilization with smooth induction and recovery phases [48]. The higher dose consistently produced good muscle relaxation without pain upon injection, providing adequate anesthesia for minor surgical procedures of short duration [48]. Behavioral changes observed during induction included sneezing, moderate hypothermia, apneustic breathing, paddling, and swimming motions [49].

Drug combinations using TZ may improve the duration and quality of anesthesia and analgesia (Table 3) [14,43,45,49,50].

### 4.4. Dosage and Clinical Application in Birds

In Common Buzzards, an oral dose of 80 mg/kg enabled stress-free capture and handling approximately 30 min post-administration, but did not induce surgical anesthesia [51]. In Japanese quails, doses ranging from 10 to 100 mg/kg, whether alone or combined with sedative drugs, produced dose-dependent sedation without anesthesia [52,53]. For non-invasive procedures, a dose up to 30 mg/kg was suggested [53]. In Pekin ducks, a dose of 13 mg/kg allowed repeated surgical liver biopsies without perioperative death [54]. A dose of 5 mg/kg IM provided immobilization of adult King Penguins for routine health examination and minor procedures for one hour, but did not reach surgical depth of anesthesia. Transient apnea was noted but no severe side effects were observed [52,55].

In ratites, species such as Emu, Rhea, and Ostrich were anesthetized with TZ alone (2.3 to 4.9 mg/kg IV) or with xylazine, butorphanol, and isoflurane for surgery [56].

For exotic pet birds, limited data exist, with sedative or short-time anesthesia dosages ranging from 10 to 30 mg/kg IM, causing possible hypothermia, salivation, and vomiting [9,57,58]. Pigeons required 40 to 60 mg/kg IM [9]. Recovery after TZ alone could be prolonged with agitation and wing flapping; no studies on combined sedation protocols are available [58,59].

### 4.5. Dosage and Clinical Application in Reptiles

Few data are available on the use of TZ in reptiles (Table 4) [60,61,62,63,64,65]. A major advantage of TZ in reptiles is the relatively low volume of injection required and its ability to sedate very large species [66]. At some dosages in some species, complete anesthetic recovery can be prolonged, sometimes taking more than 12 h [60,61,62,63,64,65,67,68,69,70,71]. Prolonged recoveries must be considered.

Regarding American alligators, TZ at 15 mg/kg IM has been successfully used for capture, transport and minor medical procedures in young American alligators [72].

In snakes, sedation or anesthetic induction are recommended at low doses (2–5 mg/kg) to manage fractious snakes and facilitate handling, sampling, and minor surgical procedures [66,70]. In one study in the Boa constrictor, TZ was associated with transient increases in heart and respiratory rates that were not associated with changes in minute ventilation, systolic blood pressure, or arterial oxygen saturation [64].

In tortoises, very few data are available. Doses up to 88 mg/kg in three-toed box turtles produced sedation without surgical depth of anesthesia [73]. Various doses between 5 and 10 mg/kg allowed sedation for physical examination, non-invasive procedures, and sometimes surgical depth of anesthesia in a dose-dependent manner. Most of the time, TZ has been shown to induce cardiopulmonary changes in reptiles similar to mammals [74,75]. Some effects on biological parameters have been described, but no clinical abnormalities or major side effects were reported [60,61,65].

**Table 4:** Use of Tiletamine–Zolazepam in reptiles.

Species and References	Anesthetic Protocols	Effects and Comments
Large pythons [68,75]	TZ 2–5 mg/kg IM	Sedation then intubation possible 45 min later
Large pythons [68,75]	TZ 4–8 mg/kg IM	Sedation to facilitate handling
Ball pythons [60]	TZ 3 mg/kg IM	Short-term anesthesia Loss of righting reflex after a mean period of 44.5 min Complete loss of all reflexes after a mean period of 51 ± 13.77 min with a mean duration to regain righting reflex of 22 ± 11.08 min Allow handling, minor procedures Righting reflex return after a mean period of 94.4 ± 0.0769.12 min.
Pythons and Boas [62]	TZ 15–30 mg/kg IM	Sedation for echocardiographic examination on aggressive or agitated animals Muscular relaxation Diminution, or complete abolition, the righting reflex
Boa constrictor [64]	TZ 12.5 mg/kg IM	Safe immobilization, no surgical anesthesia
Corn Snake [74]	TZ 20 mg/kg IM	Anesthesia induction, allowing tracheal intubation and maintain using isoflurane surgery possible
Green Iguana [65]	TZ 6.3–14.7 mg/kg IM	Surgical anesthesia Quick induction, muscle relaxation orotracheal intubation Long recovery (>45 min for some patients)
Green Iguana [63,69]	TZ 7.5 mg/kg IM	Surgical anesthesia for exploratory coeliotomy
Central Bearded Dragons [61]	TZ 20 mg/kg IM or SC	Deep sedation–light anesthesia for clinical procedures SC route less effective than IM IM: jaw tone lost (90%), loss of righting reflex prolonged recoveries but similar between routes (mean 57–69 min) IM route: a deeper and more consistent plane of sedation Prolonged recoveries

Abbreviations: IM = Intramuscularly, SC = Subcutaneous, TZ = Tiletamine–Zolazepam.

## 5. Clinical Application in Wildlife Species

TZ pharmacokinetic data are scarce in wildlife species. Notwithstanding the inherent variability among wildlife species, anesthetic protocols share fundamental requirements. These include the prioritization of agents with broad therapeutic indices and the systematic consideration of the animal’s body weight and clinical status. Remote injection is another limitation, involving the need for the smallest injection volume possible and a variety of routes of injection [76,77]. Rapid induction and full or partial antagonism are advisable to facilitate capture in the field and a fast and smooth recovery. TZ complies with most of the requirements listed above and as such has been used alone or in combination with other drugs in wildlife [9,78]. Prolonged anesthetic recovery is the main concern after TZ in wildlife anesthesia. Advantages of TZ and alpha-2 agonist combinations include partial reversibility of the anesthesia protocol at the end of the procedure. Flumazenil has also been used to antagonize zolazepam in an attempt to hasten recovery. However, the effect seems to be species-specific. For example, the effects of flumazenil on recovery times in bonobos could not be confirmed, whereas in cheetahs, recovery was significantly shorter and smoother when flumazenil had been administered [79,80]. On the contrary, flumazenil did not have any effect on the recovery time of white-tailed deer (*Odocoileus virginianus*) anesthetized with TZ alone [81]. Wildlife species can be potentially dangerous, and the unexpected recovery of some patients can present a significant safety issue for the people around them. TZ in addition to or instead of ketamine in multimodal anesthesia protocols seems to decrease the risk of sudden arousal in bears and primates [79,82,83]. Drawing upon published literature and the authors’ clinical experience with large wildlife, the lyophilized formulation of TZ permits the preparation of high-concentration solutions and facilitates reconstitution or admixture with other agents, such as ketamine or alpha-2 agonists. Within the framework of balanced anesthesia, these concentrated combinations minimize total injectate volumes. This is particularly advantageous for remote delivery via dart (typically 3–5 mL) in large-bodied species or for manual administration in captive wildlife under behavioral restraint [82,84].

### 5.1. Dosage and Clinical Application in Primates

The recommended dosage for TZ in non-human primates in sanctuaries ranges from 1 to 15 mg/kg, with no life-threatening side effects reported even at higher dosages [85,86]. While tiletamine–zolazepam (TZ) is favored in great ape anesthesia for its high potency and low injection volume, its specific hemodynamic effects remain poorly characterized. Although early primate studies suggested stable cardiopulmonary parameters [87], these findings lack corroboration from dedicated pharmacodynamic research in great apes. Given the inconsistent clinical reports and the high prevalence of cardiovascular disease as a primary cause of mortality in these taxa, the cardiovascular stability of TZ-based protocols should be interpreted with caution. As a sole anesthetic agent, TZ has been used at dosages from 3 to 6.6 mg/kg in chimpanzee (*Pan troglodytes* (Table 5) [88]. In great apes, lower doses of TZ were administered when in combination with medetomidine compared to TZ alone (Table 5) [79,87,88].

In mature wild ring-tailed lemur (*Lemur catta*), duration of anesthetic recovery is correlated with total TZ dose [89].

### 5.2. Dosage and Clinical Application in Wild Felines

Historically, the tiger (*Panthera tigris*) was identified as the only species in which TZ induced adverse effects; specifically, delayed post-recovery neurological disorders were reported in approximately 8.6% of cases [90,91]. Although TZ is not strictly contraindicated in tigers, clinical guidelines emphasize conservative dosing and the maintenance of optimal hydration [91]. Long-term clinical data and the authors’ clinical experience further support the application of TZ protocols in this species. Notably, a recent retrospective study found no significant difference in the risk of respiratory depression and seizures between tigers immobilized with TZ–medetomidine versus ketamine–medetomidine. However, the administration of TZ as a sole agent is more likely to correlate with a higher incidence of ataxia during the recovery phase [92]. This suggests that while the acute seizure risk is low, the potential for delayed neurological effects warrants continued clinical vigilance. Furthermore, the administration of TZ in tigers with renal insufficiency is generally cautioned against, as observed post-recovery neurological disorders may result from the protracted elimination of tiletamine or its active metabolite [93].

When used alone, TZ anesthesia and recovery time are significantly longer than anesthesia with a combination of medetomidine and ketamine in Sunda clouded leopards (*Neofelis diardi)* (Table 6) [94]. The implementation of balanced anesthesia, incorporating agents such as alpha-2 agonists, is frequently employed to reduce the required dose of TZ. This multimodal approach effectively decreases the total drug volume required and facilitates a more rapid anesthetic recovery.

TZ–medetomidine (TZM) allowed safe and successful immobilization of African lions (*Panthera leo*) with low doses of TZ in combination with medetomidine (Table 6) [82,95,96]. TZM and medetomidine-ketamine result in similar anesthetic recovery times in captive Amur leopard cats (*Prionailurus bengalensis euptailurus*) [97]. In leopards (*Panthera pardus*), the co-administration of medetomidine permits a reduction in the required dose of TZ compared to monotherapy. While the metabolism of the molecules and renal status may influence excretion across *Panthera* species, recent evidence identifies no justification for the contraindication of TZ in the chemical restraint of *Panthera* spp. [92,93] (Table 6).

In the authors’ clinical experience, effective anesthesia in adult Amur tigers (*Panthera tigris altaica*) can be achieved using a multimodal protocol. This regimen consists of pre-anesthetic sedation with midazolam (0.036 mg/kg) and medetomidine (0.018 mg/kg), followed by induction with TZ (0.75 mg/kg), ketamine (0.75 mg/kg), butorphanol (0.045 mg/kg), and methadone (0.045 mg/kg).

### 5.3. Dosage and Clinical Applications in Bears

The pharmacokinetics of TZ have been reported in polar bears (*Ursus maritimus*). Reported half-life (mean ± SE) of TZ in polar bears is 1.8 ± 0.2 h for tiletamine and 1.2 ± 0.08 h for zolazepam [98].

In captive and wild bear species, TZ is one of the most used anesthetics. In combination with alpha-2 agonists, TZ doses of 2–6 mg/kg have been used in Brown bears (*Ursus arctos*) compared to 10 mg/kg of TZ alone in Polar bears [99].

In captive settings (zoo), a combination of TZ (1.25 mg/kg) and medetomidine (0.020–0.025 mg/kg) has been effectively utilized for European brown bears (*Ursus arctos arctos*), based on the authors’ clinical experience. Similarly, based on personal clinical experience (CG), adult captive polar bears (*Ursus maritimus*) were anesthetized for dental examinations and crating, utilizing a multimodal intramuscular admixture. This admixture consisted of TZ (0.6–0.8 mg/kg), ketamine (0.6 mg/kg), medetomidine (0.02 mg/kg), methadone (0.04 mg/kg), butorphanol (0.04 mg/kg), and midazolam (0.04 mg/kg). To maintain anesthesia depth, supplemental ketamine (0.6 mg/kg, IM) was administered 20–25 min post-induction. Reversal was performed 70–90 min after induction using atipamezole (0.1 mg/kg) and naltrexone (0.25 mg/kg) administered intramuscularly.

Naltrexone was administered to antagonize butorphanol and facilitate rapid recovery. Due to its longer half-life compared to naloxone, naltrexone significantly reduces the risk of renarcotization. Furthermore, its availability as a veterinary-labeled product offers superior clinical and logistical accessibility compared to human-use naloxone formulations.

### 5.4. Dosage and Clinical Applications in Wild Ungulates

In free-ranging warthogs (*Phacochoerus africanus*), the volume of ketamine required to anesthetize an adult animal would be impractical, whereas the volume of a mixture of TZ–ketamine–medetomidine–butorphanol could fit into a 3 mL dart (Table 7) [76].

In Eurasian wild boar (*Sus scrofa*), the anesthetic protocol associating TZ and medetomidine combines high efficiency and low volumes of drug, which is valuable for the teleanesthesia of large wild suids (Table 7) [100,101]. One of the authors frequently uses TZ lyophilizate diluted with ketamine for the anesthesia of large ungulates and cervids.

Adult male American bison (*Bison bison*) are successfully immobilized with a mixture of TZ–ketamine–medetomidine–butorphanol (TZKMB) fitting in a 5 mL dart.

In wild equids, TZ–medetomidine–butorphanol (TZMB) offers an alternative to etorphine and is usually recommended to achieve fast immobilization (Table 7) [78,84].

For the immobilization of subadult and adult American bison (*Bison bison*) maintained in large outdoor enclosures, the authors’ clinical experience supports a remote-delivery protocol administered via darting. Successful induction in group-housed individuals has been achieved using a combination of tiletamine–zolazepam (200–300 mg), ketamine (200–300 mg), medetomidine (6–12 mg), and butorphanol (20–30 mg). This regimen is particularly effective for managing the logistical challenges of social groups in expansive environments.

### 5.5. Dosage and Clinical Application in Wild Avian Species

In ratites (palaeognathae), anesthesia protocols that include TZ offer an alternative to the use of ultrapotent opioids. However, when TZ is used alone, struggling is described during the recovery phase, and additional midazolam or diazepam can be included in the induction protocol to mitigate these adverse effects [102]. As in mammalian species, the combination of TZ and alpha-2 agonist is also recommended in Ostrich [103]. In Struthioniformes, TZ has been used in ostrich and rhea and provides a safe and reliable anesthesia with good muscle relaxation without prolonged recovery time [102,104].

### 5.6. Dosage and Clinical Application in Wild Reptiles

In giant tortoises (*Chelonoidis becki*), TZ (10 mg/kg) provides enough muscle relaxation and sedation to allow non-invasive procedures with a relatively short recovery time if the patient is maintained at the upper range of the preferred optimal temperature zone [105].

### 5.7. Dosage and Clinical Applications in Marine Mammals

TZ has been used IM with good success in southern elephant seals at a dose of 1 mg/kg as well as in many Otariid species at a dose of 1.5 to 2.0 mg/kg [106].

Californian elephant seal pups (approximately 80 kg bodyweight) were successfully sedated using TZ at 1 mg/kg IM in combination with Medetomidine at 0.07 mg/kg [107].

## 6. Conclusions and Future Directions

The TZ combination is frequently utilized in veterinary anesthesia due to its synergistic pharmacological properties and rapid onset of action. The interaction between the dissociative agent tiletamine and the benzodiazepine zolazepam provides a predictable anesthetic plane applicable across diverse clinical settings. In the context of exotic and wildlife species, TZ remains a notable component of anesthetic protocols, largely due to its lyophilized formulation, which facilitates the low-volume administration required for remote delivery in non-domesticated taxa. However, the clinical application of TZ must be balanced against its inherent limitations, including significant interspecies variability in metabolism and the lack of a specific antagonist.

The clinical application of TZ in exotic and wildlife species necessitates the proactive anticipation and management of intraoperative complications, which vary significantly across taxa and anesthetic protocols. Respiratory depression, hypoventilation, and hypoxemia are primary concerns, particularly when TZ is administered in combination with other sedative agents. Consequently, oxygen supplementation is strongly recommended whenever feasible, and assisted ventilation should be initiated in the event of apnea or severe hypoventilation. Cardiovascular stability may also be compromised depending on the specific dosage and drug combinations employed. Continuous monitoring of heart rate and, where technically possible, blood pressure is advised. Standard supportive measures, including tailored fluid therapy and the titration of anesthetic agents, should be implemented as required. Prolonged or dysphoric recovery is a well-documented challenge associated with TZ. The integration of multimodal protocols, such as the inclusion of alpha-2 agonists, may enhance recovery quality. Furthermore, the use of partial reversal agents should be considered when clinically appropriate. In wildlife and potentially hazardous species, incomplete anesthesia or unexpected arousal poses a critical safety risk to both the patient and the veterinary team. Precise dosing, optimized drug combinations, and vigilant monitoring are essential to mitigate these risks. Additionally, hypothermia remains a frequent complication, particularly in small exotic species; therefore, active thermal support must be maintained throughout the perioperative period to ensure physiological stability. Ultimately, the successful use of TZ-based protocols depends on the anticipation of species-specific risks and the implementation of rigorous monitoring and supportive interventions to ensure safe anesthetic outcomes.

It is imperative to recognize that each anesthetic situation is unique. Consequently, the selection of anesthetic protocols should integrate the principles of evidence-based medicine (EBM) with clinical expertise and the specific physiological and clinical status of the animal. This review comprises a comprehensive analysis of existing literature complemented by a critical assessment derived from the authors’ clinical experience.

Contemporary clinical practice increasingly emphasizes multimodal, balanced anesthesia. The integration of TZ, frequently in combination with alpha-2 adrenoceptor agonists or other sedative agents, is intended to improve the quality of the recovery period and enhance analgesia.

Robust clinical and pharmacokinetic data support the widespread use of TZ-based protocols in several mammalian groups, including various non-human primates, bear species, and wild felids. In these taxa, TZ is a recognized component of balanced anesthesia, frequently combined with alpha-2 agonists to ensure reliable induction and manageable recovery.

Conversely, the evidence base remains more limited for some other specialized groups. While reports exist for certain exotic pet birds and reptiles, these data often highlight challenges such as unpredictable recovery times and inconsistent anesthetic depth. In these species, as well as in specific rodents, TZ must be administered with caution, utilizing rigorous physiological monitoring and individualized risk assessment to ensure animal safety.

TZ-based protocols should be guided by a critical appraisal of available data, the specific physiological status of the animal, and a commitment to advancing evidence-based protocols through continued controlled research. Despite being available for approximately 40 years, TZ combination remains the subject of numerous publications, which underscores its ongoing relevance and the continued interest in refining its application.

Further research exploring optimized protocols, particularly for specific patient populations or in combination with other agents, is warranted to refine its use and maximize patient safety. From a practical perspective, the guidance presented herein provides a framework for practitioners to optimize anesthetic management in exotic pet species and wildlife species, supporting a judicious approach to TZ-based protocols. In conclusion, this review can serve as a resource for the veterinary practitioner, supporting the effective use of TZ-based protocols for informed, evidence-based decision-making.

## Figures and Tables

**Table 1:** Use of Tiletamine–Zolazepam in rabbits.

Drugs and Dosages	Effects and Comments
TZ 15 mg/kg IM TZ 15 mg/kg + X 5 mg/kg (TZX) IM [17]	TZX: satisfactory anesthetic effect Duration of anesthesia and loss of reflexes significantly longer in the TZX group (mean recovery time of 115.50 ± 9.26 min (TZX)/61.15 ± 6.95 min in (TZ)) Pedal withdrawal reflex of the pelvic limb remained present in the TZ group Respiratory rate and body temperature decreased and heart rate reduced only in the TZX group
TZ 10 mg/kg IN [20]	mean onset time: 2.5 ± 1.13 min mean duration: 44.4 ± 12.73 min significant decrease in respiratory rate and a decrease in hemoglobin saturation
TZ 15 mg/kg + B 0.5 mg/kg IN or IM [15]	Routes of administration had no significant effect on the intraocular pressure and tear secretion Time to onset of sedation earlier in the IN group Mean duration of sedation longer IM (57.43 ± 3.41 min)/IN (45.0 ± 1.91 min) No difference in the sedation score
TZ 20 mg/kg IM TZ 20 mg/kg + X 3 mg/kg (TZX) IM [16]	TZ: no suppression of reflexes TZ and TZX: decreased body temperature and pH; increased HCO_3_^−^ TZ: mean arterial blood pressure and PaCO_2_ less affected/TZX TZX: reflexes absent, surgical anesthesia 60–90 min (confirmed by EEG measures) TZX: supplementation with oxygen
TZ 15 mg/kg + X 5 mg/kg IM [19]	successful surgical anesthesia (mean time 72 ± 8 min)

Abbreviations: IM = Intramuscularly, IN = Intranasal, TZ = Tiletamine–Zolazepam, X = Xylazine, B = Butorphanol.

**Table 3:** Use of Tiletamine–Zolazepam in ferrets.

Species and References	Anesthetic Protocols	Effects and Comments
Ferret [14,50]	Protocol 1 TZ 1.5 mg/kg + X 1.5 mg/kg IM Protocol 2 TZ 3 mg/kg + X 3 mg/kg IM Protocol 3 TZ 1.5 mg/kg + X 1.5 mg/kg + B 0.2 mg/kg IM	Lateral recumbency within 2 min. Similar heart rates and times from dorsal recumbency to standing Protocol 1: Endotracheal intubation not possible Protocol 2: increased duration of analgesia, endotracheal intubation possible Protocol 3: longer durations of analgesia (mean 90.0 min) and endotracheal intubation (mean 84.8 min) Systolic blood pressure and ventilatory function lowered, short period of hypoxia (insufflation is recommended) Preferred protocol for anesthetic induction, immobilization, and reliable analgesia smooth recovery

Abbreviations: IM = Intramuscularly, TZ = Tiletamine–Zolazepam, X = Xylazine, B = Butorphanol.

**Table 5:** Use of Tiletamine–Zolazepam in primates.

Species and References	Anesthetic Protocols	Comments
Chimpanzee (*Pan troglodytes*) [88]	TZ 3–6.6 mg/kg	Sole anesthetic agent
Chimpanzee (*Pan troglodytes)* (author individual experience)	TZ 1.5–2 mg/kg + M 0.02–0.03 mg/kg + K 1–2 mg/kg IM	Balanced anesthesia
Great apes [79,88]	TZ 6–10 mg/kg TZ 2 mg/kg + M 0.02–0.03 mg/kg	
Ring-tailed lemur (*Lemur catta*) [89]	TZ 12 ± 5 mg/kg (mature lemurs) TZ 19 ± 7 mg/kg (young lemurs)	Initial dose for young lemurs significantly higher than dose for mature lemurs

Abbreviations: IM = Intramuscularly, TZ = Tiletamine–Zolazepam, M = Medetomidine, K = Ketamine.

**Table 6:** Use of Tiletamine–Zolazepam in wild felines.

Species and References	Anesthetic Protocols
Tiger (*Panthera tigris*) [91]	TZ 0.8–1.2 mg/kg + M 0.018–0.024 mg/kg
Sunda clouded leopards (*Neofelis diardi)* [94]	TZ 6.5–10 mg/kg
African lion (*Panthera leo*) [95,96]	TZ 0.6 mg/kg + M 0.03–0.07 mg/kg
African lion (*Panthera leo*) [41]	TZ 80 mg + M 6 mg as total dose for subadult and adult lions IM Reversal with atipamezole 15 mg total dose IM
Leopards (*Panthera pardus*) [93]	TZ 5–6 mg/kg TZ 2–3 mg/kg + M 0.02–0.03 mg/kg

Abbreviations: IM = Intramuscularly, TZ = Tiletamine–Zolazepam, M = Medetomidine.

**Table 7:** Use of Tiletamine–Zolazepam in wild ungulates.

Species and References	Anesthetic Protocols
free-ranging warthogs (*Phacochoerus africanus*) [76]	TZ 0.69 ± 0.15 mg/kg + K 1.43 ± 0.21 mg/kg + M 0.07 ± 0.01 mg/kg + B 0.26 ± 0.04 mg/kg
Eurasian wild boar (*Sus scrofa*) [100]	TZ 3 mg/kg + M 0.05 mg/kg
Wild equids [84]	TZ 2.72 mg/kg + M 0.08 mg/kg + B 0.19 mg/kg
Feral horse (*Equus caballus*) [78]	TZ 3.61 mg/kg + M 0.15 mg/kg

Abbreviations: TZ = Tiletamine–Zolazepam, M = Medetomidine, B = Butorphanol, K = Ketamine.

## Data Availability

No new data were created or analyzed in this manuscript. Data sharing is not applicable to this article.
